# A top-down measure of gene-to-gene coordination for analyzing cell-to-cell variability

**DOI:** 10.1038/s41598-021-90353-w

**Published:** 2021-05-26

**Authors:** Dana Vaknin, Guy Amit, Amir Bashan

**Affiliations:** Physics Department, Bar-Ilan University, Ramat Gan, Israel

**Keywords:** Complex networks, Gene regulatory networks, RNA sequencing, Dynamic networks

## Abstract

Recent technological advances, such as single-cell RNA sequencing (scRNA-seq), allow the measurement of gene expression profiles of individual cells. These expression profiles typically exhibit substantial variations even across seemingly homogeneous populations of cells. Two main different sources contribute to this measured variability: actual differences between the biological activity of the cells and technical measurement errors. Analysis of the biological variability may provide information about the underlying gene regulation of the cells, yet distinguishing it from the technical variability is a challenge. Here, we apply a recently developed computational method for measuring the global gene coordination level (GCL) to systematically study the cell-to-cell variability in numerical models of gene regulation. We simulate ‘biological variability’ by introducing heterogeneity in the underlying regulatory dynamic of different cells, while ‘technical variability’ is represented by stochastic measurement noise. We show that the GCL decreases for cohorts of cells with increased ‘biological variability’ only when it is originated from the interactions between the genes. Moreover, we find that the GCL can evaluate and compare—for cohorts with the same cell-to-cell variability—the ratio between the introduced biological and technical variability. Finally, we show that the GCL is robust against spurious correlations that originate from a small sample size or from the compositionality of the data. The presented methodology can be useful for future analysis of high-dimensional ecological and biochemical dynamics.

## Introduction

Biological functions within the cell are carried out by gene products, such as RNA chains and proteins^1^. The high-dimensional data of the gene expression profile is thus an elemental and useful representation of the cellular activity^2^. Until recently, only bulk RNA sequencing technologies were available to study gene expression patterns at the population level. Such measurements reflect the averaged gene expression across thousands of cells^3,4^. The recent development of single-cell RNA sequencing (scRNA-seq) technologies allow the dissection of gene expression at single-cell resolution^5–9^. A central observation from such single-cell measurements is that, even within populations of cells from the same tissue and of the same cell-type, the gene expression profiles substantially deviate across different individual cells.

This cell-to-cell variability may arise from two fundamentally different types of processes: (1) processes that happen while the cell is still alive and active, and (2) processes that take place while the cell content is dissected and measured. Accordingly, the cell-to-cell variability that stems from such processes can be termed ‘biological’ or ‘technical’ variability, respectively. Biological variability is the result of various sources, such as, inherent stochasticity in the biochemical process of gene expression^10–12^, random genetic and epigenetic mutations in different individual cells^13,14^, differences in the internal states in the cell cycle progression^15,16^, or sub-populations of cells owing to subtle environmental differences or cell-subtypes^17–19^. In contrast, technical variability represents statistical and measurement limitations of the single-cell sequencing procedure, e.g., sampling noise in the genetic survey and stochastic over- and under-amplification of random genes^20,21^. Naively, measuring cell-to-cell variability by evaluating the dissimilarity between the measured gene expression profiles of different cells does not discriminate between biological and technical variability. Since the real biological phenomena are manifested only in the biological variability and not in the technical noise, there is a practical need to be able to distinguish between these types of variability.

To address this challenge, several experimental and computational techniques have been developed. Some of the sources for biological variability can be controlled by dividing heterogeneous populations of cells into sub-populations which are homogeneous with respect to a particular factor^22–24^. For example, focusing on cells from the same cell-cycle stage or cells belonging to the same sub-type. Yet, even in such seemingly homogeneous sub-populations, biological variability still occurs from processes of stochastic nature that affect each cell differently^25^. To distinguish between biological and technical variability within such homogeneous sub-populations of cells, characteristic features of the variability types are examined^26^. A central approach considers that technical variability of individual genes follows a typical form of a variance-to-mean ratio whereas larger variability in an individual gene may indicate a biological source^27–29^. Other computational approaches assume that biological variability is typically characterized by interrelations between the expression of different genes, and thus, employ the measure of the correlation between pairs of genes to identify biological variability^30^.

The correlation-based approaches are commonly implemented in a ‘bottom-up’ fashion, e.g., by calculating co-expression matrices^31^. However, without *a priori* knowledge of the intricate map of gene-to-gene regulatory interactions, it is extremely difficult to accurately infer the interactions for a large number of genes, mainly since large calculated co-expression matrices contain a considerable amount of noise. In addition, different co-expression measures are designed to capture specific features which are not necessarily optimal for depicting all types of gene-to-gene interrelations (e.g., Pearson correlations represent only linear relationships), and are focused on pairwise interactions while an individual gene may be controlled by a combination of multiple regulators^32,33^.

Recently, a ‘top-down’ approach has been introduced to analyze scRNA-seq data by evaluating the global coordination level between genes (named GCL)^34^. Here, following the above-mentioned approaches that focus on the interrelations between genes to detect biological activity, we propose to use the GCL as an indication for the biological origins of the measured cell-to-cell variability. We systematically analyze synthetic data generated from mathematical models of gene regulatory dynamics, where biological variability is introduced as random variations between the generating models of different individual cells. We show that the GCL is an effective tool in the analysis of cell-to-cell variability in single-cell data. The GCL is not negligible wherever the gene regulatory models include interactions between the genes, and decreases as the amount of random variations in the dynamics increases. We also calculate the GCL for cells generated from models that involve both biological and technical variability, where the latter is introduced as independent measurement noise. We find that the GCL can distinguish between different assemblages of cells with the same measured cell-to-cell variability but a different ratio of biological–technical variability. Finally, we show that the GCL is robust against spurious correlations that originate from a small sample size or the compositionality of the data.

## Methodology

### Annotations used in the manuscript

In the following, we use these definitions: the gene expression of gene *i* in the mathematical model of gene regulatory dynamics is noted as $x_i$. The solution of this model, which represents the steady state or the actual gene expression of gene *i* is noted as $x^*_i$. The measured expression, which includes both the actual expression level and measurement noise, is noted as $\tilde{x}_i$.

### Global coordination level (GCL)

The GCL is a “top-down” computational method to evaluate the system-wide transcriptional multivariate dependency of genes, without inferring the whole network of pairwise correlations^34^. We refer to a set of *M* measured cells with *N* genes as a matrix $\tilde{X}_{N\times M}$, where every vector column $\tilde{\varvec{x}}^{(\nu )}$ $(\nu =1\dots M)$ represents the individual cell $\nu$, and each element $\tilde{\varvec{x}}^{(\nu )}_i$ $(i=1\dots N)$ represents the measured expression value of gene *i* in that cell. The GCL calculation is performed as follows. First, we divide the matrix $\tilde{X}_{N\times M}$ into two random complementary parts *A* and *B*, where each part contains *N*/2 rows, i.e., each part contains *N*/2 gene expression values for *M* cells. Second, we calculate the “bias-corrected distance correlation” (bcdCorr) measure^35^ on (*A*, *B*). In brief, the bcdCorr, a refined version of the “distance correlation” (dCorr) measure^36^, evaluates the level of dependence between two high-dimensional variables by testing how the distance between two samples with respect to one variable is changed compared to the distance between the same two samples with respect to other variable. Thus, the bcdCorr is a measure of the dependency level between gene-sets *A* and *B*. Finally, we repeat these two steps *m* times and define the GCL as the average bcdCorr, i.e., the GCL is defined as

1 $$\begin{aligned} \text {GCL}(X) = \frac{1}{m} \sum _{k=1}^m \text {bcdCorr}\left( A^k,B^k\right) , \end{aligned}$$

where all *m* divisions $(A^k,B^k)$ are independent. As the GCL typically stabilizes for $m > 10$, in our analysis we choose $m = 50$. For a large sample size, the empirical GCL is zero in the case of independent gene expression; whereas a significantly non-zero GCL reflects coordinated transcriptional expression, which could be interpreted as a result of underlying molecular dynamics, such as gene-to-gene regulatory interactions.

**Figure 1:**
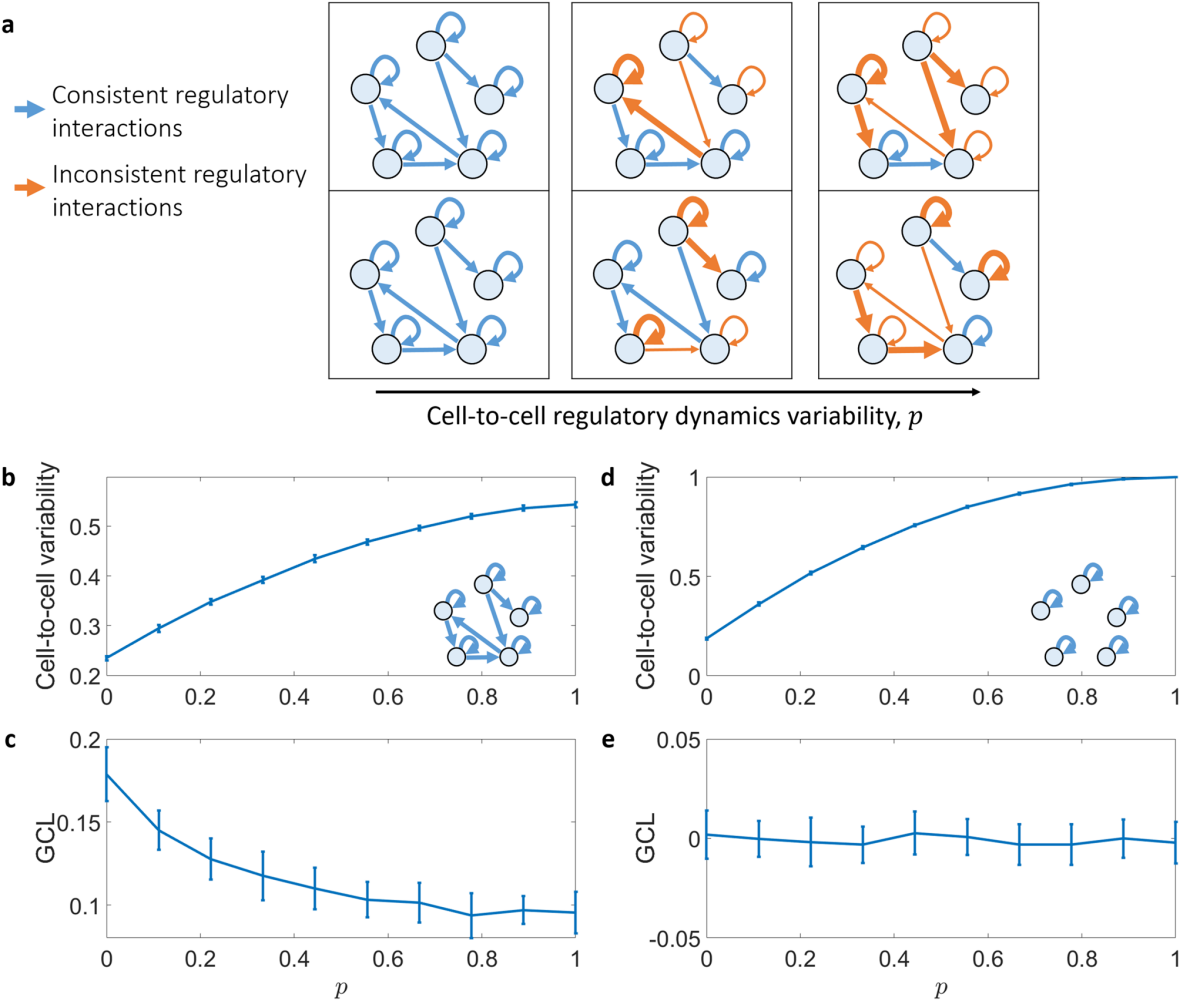
The effect of heterogeneous regulatory dynamics on the cell-to-cell variability and gene-to-gene coordination. (**a**) Schematic illustration of gene regulatory networks (GRNs) of two different cells. The weight of each gene–gene interaction and self-regulatory interaction (blue arrows) is replaced with a random value (orange arrows) with probability *p*. For $p=0$, both cells have the same GRNs. (**b**,**c**) Cell-to-cell variability and GCL score as a function of *p* for a cohort of simulated cells $X_{N\times M}^*$. The cell-to-cell variability was calculated as the average dissimilarity between all cell pairs, defined as one minus the Spearman correlation. (**d**,**e**) Same analysis as in (**b**,**c**), but with GRNs that have only self-regulation term, i.e., no gene–gene interactions. For these figures, we have $M=100$ cells and $N=200$ genes for each data point. The data points and error bars represent mean and standard deviation over 20 realizations, and every GCL score is calculated for $m = 50$ different divisions. Unlike the cell-to-cell variability, the GCL can indicate the presence of gene–gene interactions in the underlying dynamics.

### Numerical model for synthetic gene expression data

To investigate the ability of the GCL to reveal biological and technical variability in gene expression data, we apply it to synthetic cells generated from models of gene regulatory dynamics. The model simulates ‘cohorts’ of gene-expression profiles with varying levels of cell-to-cell variability that originated from both differences between the actual expression profiles (‘biological variability’) and stochastic measurement noise (‘technical variability’).

We define the vector $\varvec{x}^{*(\nu )}$ as the actual gene expression of an individual cell $\nu$. The expression profile $\varvec{x}^{*(\nu )}$ is modelled as the steady state of a set of coupled ordinary differential equations (ODEs), representing the gene regulatory dynamics^33,37,38^. Specifically, we use the following set of ODEs,

2 $$\begin{aligned} \dot{\varvec{x}}^{(\nu )}_i=-B\varvec{x}^{(\nu )}_i+\sum _{j}w^{(\nu )}_{i,j}\frac{\varvec{x}_j^{(\nu )n}}{1+\varvec{x}_j^{(\nu )n}}~. \end{aligned}$$

The first term expresses a self degradation of gene *i*. The second term is responsible for the growth of $\varvec{x}^{(\nu )}_i$ as a Michaelis–Metnten kinetics function^37^ of $\varvec{x}^{(\nu )}_j$, i.e., gene *i* is activated by gene *j*. The activation relation can be represented as a link in the gene regulatory network (GRN) with weight $w^{(\nu )}_{i,j}$ (see Fig. 1**a**). In our simulation we use GRNs with self regulation and random links between the nodes, i.e., each pair of genes are connected with a constant probability in the form of an Erdős-Rényi network with an average degree equals to two. Finally, we set $B = 1$, $n = 1$, and the GRN weights $w^{(\nu )}_{i,j}$ (for existing links) are randomly selected from the uniform distribution $\mathcal {U}(0,2)$.

We define the matrix $X^*_{N\times M}$ as a ‘cohort’ of *M* expression profiles $\varvec{x}^{*(\nu )}$ with *N* genes each. For a given GRN dynamics, defined by the matrix of weights $w^{(0)}$, we generate different expression profiles by performing two types of changes in the dynamics. First, a random subset of the genes in each cell are set as inoperative, i.e., their expression levels are set to zero. Specifically, in our simulations we randomly choose 5 out of 200 genes to be inoperative. Second, the GRN dynamics of each single-cell $\nu$, $w^{(\nu )}$ ($\nu = 1\ldots M$), is generated as random variations of $w^{(0)}$, where the GRNs’ heterogeneity is controlled by the parameter *p* (see Fig. 1**a**). Specifically, the structure of the GRNs of all cells is the same as $w^{(0)}$, i.e., $w_{i,j}^{(\nu )} \ne 0$ if and only if $w^{(0)}_{i,j} \ne 0$. The interactions weights are randomly chosen from a uniform distribution $\mathcal {U}(0,2)$ with probability *p*, otherwise $w_{i,j}^{(\nu )} = w^{(0)}_{i,j}$. The expression profile of each cell $\varvec{x}^{*(\nu )}$ is generated by solving the GRN differential equations with random initial conditions and evaluating the steady state using the ode45 MATLAB function.

Finally, we simulate measurement errors by introducing random noise to the actual gene expression profile. Mathematically, we assume a model where a measured expression value of gene *i* in an individual cell $\nu$, $\tilde{\varvec{x}}^{(\nu )}_i$ is represented as

3 $$\begin{aligned} \tilde{\varvec{x}}^{(\nu )}_i=\varvec{x}^{*(\nu )}_i\cdot \left( 1+\epsilon ^{(\nu )}_i\right) , \end{aligned}$$

where $\varvec{x}^{*(\nu )}_i$ represents the actual gene expression, and $\epsilon ^{(\nu )}_i$ represents the measurement error. The stochastic noise values $\epsilon ^{(\nu )}_i$ are generated from a normal distribution $\mathcal {N}(0,\,{\sigma }^{2})$.

To summarize, in our simulations the cell-to-cell variability of a measured cohort of cells $\tilde{X}_{N\times M}$ (of a specific $w_{0}$) is determined by two parameters: *p* and $\sigma$. Thus, the ‘biological variability’ is generated using the parameter *p* which controls the heterogeneity of the underlying regulatory dynamics, and the ‘technical variability’ is generated using the parameter $\sigma$ which controls the level of the stochastic noise.

**Figure 2:**
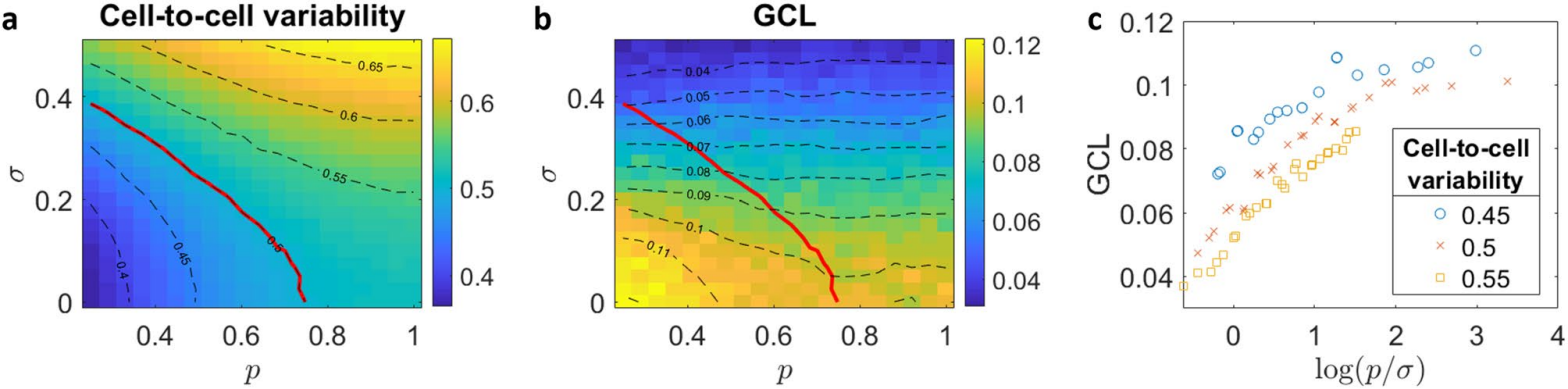
Cell-to-cell variability and GCL score as a function of heterogeneity, *p*, and technical noise, $\sigma$. (**a**) Cell-to-cell variability and (**b**) GCL heat-maps for different values of *p* and $\sigma$. Each data point represents a cohort of simulated expression profiles $\tilde{X}_{N\times ~M}$ with $M = 100$ cells and $N = 200$ genes. In both (**a**) and (**b**) contour lines are marked in black dashed lines. The contour line where the variability is equal to 0.5 is colored in red. (**c**) GCL as function of $\log (p/\sigma )$ along three contour lines of the cell-to-cell variability. In all figures, we average over 20 realizations. Every GCL score is calculated for $m = 50$ different divisions.

**Figure 3:**
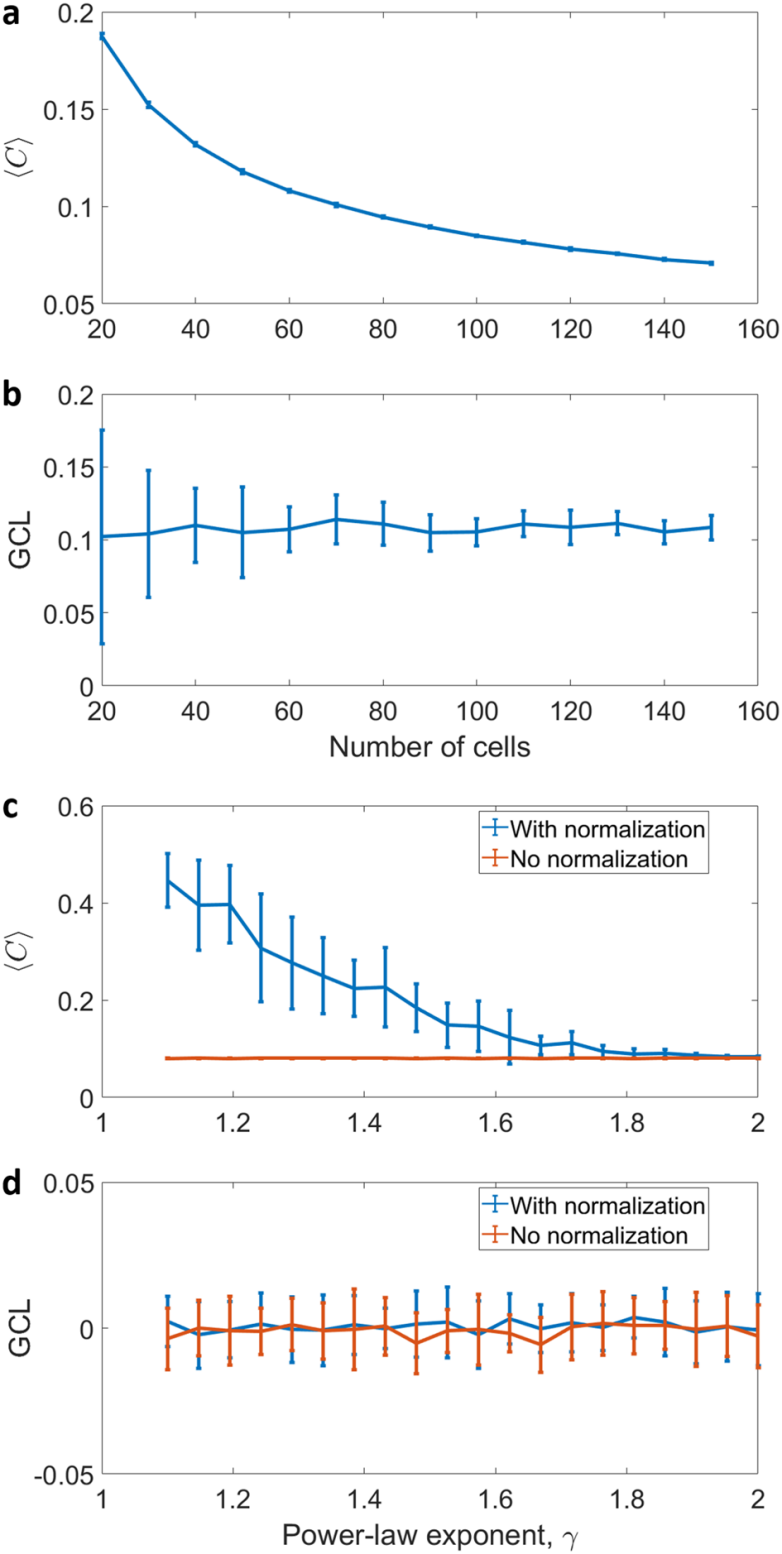
Comparison of the GCL score with the averaged co-expression $\langle C \rangle$. $\langle C \rangle$ is defined as the average of the absolute values of Spearman correlations between all the gene pairs, and thus may be used for detecting gene–gene interactions. (**a**,**b**) Simulated expression profiles $\tilde{X}_{N\times ~M}$ were created with $p = 0.5$ and $\sigma =0$, for different numbers of cells. The GCL measure identifies the coordination between the genes and, in contrast to the $\langle C \rangle$, remains stable even for a small number of cells. (**c**,**d**) Simulated compositionality in $M = 100$ expression profiles with different levels of heterogeneity, parameterized by the exponent $\gamma$ of the power-law distribution. High values of $\gamma$ correspond with more homogeneous expression profiles, whereas $\gamma \rightarrow 1$ corresponds with high heterogeneity. In this case, since the cell-to-cell variability is generated as independent random variations of the master profile in the different genes (see text), no co-expression and gene-to-gene coordination is expected. For each data point in both figures, we have $N = 200$ genes with an average of 20 realizations, and every GCL score is calculated for $m = 50$ different divisions.

## Results

We start by demonstrating that by applying GCL on a cohort of steady-states (samples), it can capture the presence of gene–gene interactions in the underlying model. This is in contrast with the cell-to-cell variability. We compare two different models for generating cohorts of samples, $X^{*}_{N\times M}$, that represent the actual gene expression of *M* cells with *N* genes, without adding measurement noise. The first model includes both self-regulation and gene–gene interaction, as detailed above in “Methodology” , while the second model has no gene–gene interactions (i.e. $w^{(\nu )}_{i,j} = 0$ for any $i\ne j$ in Eq. (2)). In both models, increasing the GRNs’ heterogeneity level, *p*, increases the cell-to-cell variability (Fig. 1b,d). This is expected as the heterogeneity reduces the similarity between the equations which regulates the different cells, leading to a larger variability in the steady states. However, contrary to the variability score, the curve of the GCL score as a function of *p* behaves differently for these two models (Fig. 1c,e). In the first model, where the GRN dynamics contains gene–gene interactions, the GCL is significantly larger than zero for small values of *p*, see Fig. 1b. In addition, as the heterogeneity level increases, the gene–gene interaction are less consistent across different cells, leading to decreased GCL. In marked contrast, where the GRN dynamics does not contain gene–gene interactions, the GCL score is around zero for any value of *p*, see Fig. 1e. These results demonstrate that the GCL can reveal essential features of the underlying regulatory dynamics (the presence of gene–gene interactions), which are not captured by the standard measure of cell-to-cell variability.

Next, we generate and analyze data with both biological and technical variability, which are determined by the parameters *p* and $\sigma$, respectively. These simulations represent the measured gene expression profiles, $\tilde{X}_{N\times M}$, described above in “Methodology”. We ask, given two cohorts with the same measured cell-to-cell variability, is it possible to differentiate between the one that was generated with a higher ratio of ‘biological’ compared to ‘technical’ variability? To address this question, we generated 420 cohorts, each of $M=100$ cells and $N=200$ genes, generated with $0.25 \le p \le 1$ and $0 \le \sigma \le 0.5$. For each generated cohort, we calculated both the cell-to-cell variability and the GCL. The heat-map in Fig. 2a shows that the cell-to-cell variability increases monotonically with each of the parameters *p* and $\sigma$, where different combinations of these values can lead to the same variability. Each dashed black contour line marks cohorts with equal variability, where the red line marks the cohorts with variability equals to 0.5, as a specific example. Fig. 2b shows the GCL values calculated for the same cohorts as in Fig. 2a, where each dashed black contour line marks cohorts with equal GCL. The red line in Fig. 2b marks the same cohorts with variability 0.5, as in Fig. 2a. Along this line, the GCL increases from the top-left, where the variability is dominated by ‘technical variability’ (high value of $\sigma$), towards the bottom-right, where the variability is dominated by ‘biological variability’ (high value of *p*). Figure 2c explicitly shows the increase of the GCL calculated along the red line with respect to $\log (p/\sigma )$. Qualitatively similar behavior is also seen for cohorts where the cell-to-cell variability is equal to 0.45 and 0.55. When comparing cohorts with the same measured cell-to-cell variability, the one with a higher ratio of biological versus technical variability has a higher GCL value. These results demonstrate the inability of the cell-to-cell variability measure alone in detecting essential features of the underlying dynamics, such as distinguishing between ‘technical’ versus ‘biological’ noise. A joint analysis by both measures, i.e., cell-to-cell variability and GCL, is recommended.

In addition, we compare the ‘top-down’ GCL and the classical ‘bottom-up’ co-expression matrix. The average of the gene co-expression matrix, is defined as $\langle C \rangle = \frac{2}{N(N-1)} \sum _{j = i+1}^{N}\sum _{i=1}^{N-1}\left| C_{i,j}\right|$, where a matrix element $C_{i,j}$ represents the Spearman correlation between gene *i* and gene *j*. These two approaches were recently compared on real transcriptomic data of aging cells^34^. There was a consistent pattern of reduced GCL values in aging cells across different cell types and different organisms. In contrast, there was no clear pattern of change of the average co-expression values in old cells compared with young cells (see SI of ref.^34^). Here, we study the effect of two typical features of real transcriptomic datasets on the ability of two approaches to reliably identify the interrelations between genes. The first feature, which is a typical scenario in currently available transcriptomic data sets, is a small sample size, i.e., the number of cells is relatively smaller compared with the number of genes and the number of possible gene–gene interactions. The second feature is the compositionality of the relative abundance of mRNAs in genomic survey data, which may lead to spurious correlations between genes^39–42^.

To study the effect of small number of cells, we first generate expression profiles $\tilde{X}_{N\times ~M}$ with $M=150$ cells and $N=200$ genes, following the same procedure detailed above in “Methodology” , with $p=0.5$ and $\sigma =0$. We then test the consistency of $\langle C \rangle$ and GCL values across cohorts with a decreasing number of cells. Fig. 3a shows that $\langle C \rangle$ becomes larger for smaller number of cells, while Fig. 3b shows that the GCL score is relatively consistent across the cohorts. Thus, we conclude that compared to the $\langle C \rangle$ score, the GCL is less affected by false correlations that appear due to a small number of cells. This simplified model demonstrates the disparity between the ‘top-down’ and the ‘bottom-up’ approaches. The averaged co-expression matrix, unlike the GCL, accumulates the noise from all matrix elements. This effect becomes especially pronounced in the case of a small sample size.

To study the effect of spurious correlations in compositional data, we generated cohorts of normalized ‘expression profiles’ with no real correlations between the genes. We generate the profiles as follows: first, we create a ‘master profile’, $y^{(0)}$, by generating for each ’gene’ a random number from a power-law distribution with an exponent $\gamma$, i.e., $p(x) \sim x^{-\gamma }$. Second, we generate $M=100$ profiles, where each profile $y^{(\nu )}$ ($\nu =1 \dots M$) is defined as $y^{(\nu )}_i=y^{(0)}_i \cdot \phi ^{(\nu )}_i$ ($i=1\dots N$) where $\phi ^{(\nu )}_i$ is a random number from a normal distribution with mean 1 and $\sigma = 0.2$. Finally, we normalize each profile to 1. For each cohort of profiles, with different values of $\gamma$, we calculate both the GCL and $\langle C \rangle$. Figure 3c,d show that the normalization procedure leads to spurious correlations between the genes for small values of $\gamma$, where the cells are more heterogeneous. In contrast, the GCL score is stable against this effect and correctly identifies that there is no coordination between the genes.

## Discussion

To study the sources of cell-to-cell variability, we adopt the GCL method that measures the coordination between genes in a top-down approach and compared it to other classical measures. We calculate the GCL for simulated gene expression profiles, with different levels of inconsistency in the gene regulatory dynamics (as biological variability) and different strengths of measurement noise (as technical variability). We demonstrate that positive GCL values reflect the effect of interactions between the genes. We show that in the case where the variability stems only from inconsistent dynamics across the cells, the GCL decreases as the inconsistency level of gene–gene interactions increases. However, in the case of both inconsistent dynamics and measurement noise, we show that for cohorts with the same measured variability, the one with the lower GCL has lower biological variability (and higher measurement noise) compared to the other cohort.

These results may have practical applications when comparing different data-sets for studying the source of the variability. A common task in biological experiments is to compare the gene expression between two states, e.g. control vs. disease or before and after perturbation. The GCL measure allows the detection of changes in the underlying regulatory dynamics even when the mean expression values and the cell-to-cell variability do not change.

Even with the recent explosion in available transcriptomic data, we are still far away from having a sufficiently large sample size compared with the huge number of genes. Thus, in order to get a detailed description of the complex network of gene interaction in the cells, we have to be creative in our analysis. A top-down approach indeed ignores the details of the delicate interactions, but as shown here, the GCL has considerable advantages. It can help us analyze the variability source and it is robust against spurious correlations that originate from a small sample size or the compositionality of the data. We propose the GCL measure as a new tool, which we believe can provide additional insights to the classical bottom-up approach.

### Practical guidelines for applying the GCL method in single-cell analysis

As discussed in this manuscript, the GCL method intends to analyze seemingly homogeneous cohorts of single-cell transcriptomes, where the measured variability stems mainly from intrinsic biological variability or technical errors. Here we suggest several pre-processing steps that are recommended to ensure that the analyzed cohorts are as homogeneous as possible, followed by a discussion of possible interpretations of the GCL outcomes.

When analyzing a set of expression profiles, the first step would be to reduce the heterogeneity by focusing on sub-populations of cells according to available metadata or specific transcriptional signatures. For example, three cell types that were isolated from a mixed population of hematopoietic stem cells using the expression of specific markers^43^, were analyzed separately by the GCL in ref.^34^. Another example for cell filtering is to reduce heterogeneity that stems from the cell cycle by selecting non-cycling cells or cells belonging to the same phase. A second step would be to reduce heterogeneity by performing unsupervised clustering analysis to the expression profiles and selecting only cells that belong to the same cluster or ‘sub-type’. A third step is to remove outliers, i.e., cells for which their expression profile is extremely different from the average cell. A possible outlier filtering is to calculate the average profile and all distances between it and each expression profile, and then to remove cells for which the distance from the average profile is more than two standard deviations larger than the mean distance. Finally, the GCL is also susceptible to the presence of cell-pairs with very similar profiles. In real transcriptomic data, this could be due to cells that were divided just before the moment of measurement. A simple way to filter out those cell-pairs would be to calculate cell-to-cell distances between all cell pairs and remove one of these cells if the distance is exceptionally small.

After these steps, the GCL may be interpreted as follows. When applied on a single cohort, a significant positive GCL value reflects the effect of interactions between the genes. The significance, in this case, can be evaluated by performing a Jackknife procedure on the real data and compare the results to shuffled data where the effects of interactions between genes are removed.

When two cohorts are compared, the GCL values should be investigated with regards to the measured cell-to-cell variability of these cohorts. If the variability is preserved but the GCL is different, this may indicate that the underlying dynamics in the cohort with the higher GCL are more consistent across cells. For example, in ref.^34^, reduced GCL values were found to be associated with aging cells and with increased genetic mutational load, and were interpreted as random aberrations of the cellular regulatory mechanisms. However, if the variability is not the same, the GCL should be interpreted with more caution. If the lower GCL is measured in the one with the higher variability, then the GCL may provide no additional insights. This is because both increased biological variability and increased technical variability lead to lower GCL (as shown in Figs. 1 and 2 in our manuscript). But, if the GCL difference is in the opposite direction, i.e., a lower GCL is measured in the cohort with the lower variability, this may indicate a lower coordinated biological activity compared with the other cohort.

## Data Availability

The custom MATLAB code for computing the GCL that was used in this study is available at https://github.com/guy531/gcl.
